# HIV-1-Specific CD11c^+^ CD8^+^ T Cells Display Low PD-1 Expression and Strong Anti-HIV-1 Activity

**DOI:** 10.3389/fimmu.2021.757457

**Published:** 2021-10-15

**Authors:** An-Liang Guo, Jin-Fang Zhao, Lin Gao, Hui-Huang Huang, Ji-Yuan Zhang, Chao Zhang, Jin-Wen Song, Ruo-Nan Xu, Xing Fan, Ming Shi, Yan-Mei Jiao, Fu-Sheng Wang

**Affiliations:** ^1^ Department of Immunology, School of Basic Medical Sciences, Cheeloo College of Medicine, Shandong University, Jinan, China; ^2^ Senior Department of Infectious Diseases, Fifth Medical Center of Chinese PLA General Hospital, National Clinical Research Center for Infectious Diseases, Beijing, China; ^3^ Department of Microbiology & Infectious Disease Center, School of Basic Medical Science, Peking University Health Science Center, Beijing, China

**Keywords:** HIV-1, CD11c^+^ CD8^+^ T cells, immune activation, immune exhaustion, cytotoxicity

## Abstract

Exhaustion of HIV-1-specific CD8^+^ T cells prevents optimal control of HIV-1 infection. Identifying unconventional CD8^+^ T cell subsets to effectively control HIV-1 replication is vital. In this study, the role of CD11c^+^ CD8^+^ T cells during HIV-1 infection was evaluated. The frequencies of CD11c^+^ CD8^+^ T cells significantly increased and were negatively correlated with viral load in HIV-1-infected treatment-naïve patients. HIV-1-specific cells were enriched more in CD11c^+^ CD8^+^ T cells than in CD11c^-^ CD8^+^ T cells, which could be induced by HIV-1-derived overlapping peptides, marking an HIV-1-specific CD8^+^ T cell population. This subset expressed higher levels of activating markers (CD38 and HLA-DR), cytotoxic markers (granzyme B, perforin, and CD107a), and cytokines (IL-2 and TNF-α), with lower levels of PD-1 compared to the CD11c^-^ CD8^+^ T cell subset. In vitro analysis verified that CD11c^+^ CD8^+^ T cells displayed a stronger HIV-1-specific killing capacity than the CD11c^-^ counterparts. These findings indicate that CD11c^+^ CD8^+^ T cells have potent immunotherapeutic efficacy in controlling HIV-1 infection.

## Introduction

Although antiretroviral therapy (ART) efficiently suppresses replication of human immunodeficiency virus-1 (HIV-1), it cannot eradicate viral reservoirs, resulting in viral rebound when ART is interrupted (1). Moreover, individuals living with HIV-1 infection receiving ART experience non-infectious complications and comorbidities, such as cardiovascular disease, neurocognitive impairment, and non-acquired immune deficiency syndrome malignancies due to the inflammatory response associated with HIV-1 infection (2). Identification of new targets for therapeutic intervention of HIV-1 infection is needed.

Secretion of cytokines, such as interleukin-2 (IL-2), interferon-gamma (IFN-γ), and tumor necrosis factor alpha (TNF-α), and cytotoxicity mediated by perforin, granzyme B, and CD107a are the main functional attributes of CD8^+^ T cells that exhibit anti-HIV-1 activity (3–5). Moreover, HIV-1-specific CD8^+^ T cells have a higher ability for cytokine production, antigen sensitivity, and cytotoxicity against HIV-1-infected target cells (4, 6, 7). These various functions of HIV-1-specific CD8^+^ T cells coincide with a decline in plasma viremia (8). Higher frequencies of HIV-1-specific CD8^+^ T cells are maintained in HIV-1-infected long-term non-progressors than in typical progressors (7). Collectively, these results suggest that CD8^+^ T cells are an important and attractive target because of their specific cytotoxicity against HIV-1-infected cells. However, persistent antigen stimulation is responsible for CD8^+^ T cell exhaustion associated with functional hyporesponsiveness in chronic viral infections and cancer (9, 10). Exhaustion is a progressive process involving elevated expression of immune checkpoint receptors, such as programmed cell death 1 (PD-1), T cell Ig and ITIM domain (TIGIT), and lymphocyte-activation gene 3 (LAG-3), which are linked to clinical disease progression (11). Significantly upregulated expression of PD-1 on HIV-1-specific CD8^+^ T cells has been correlated with impaired HIV-1-specific CD8^+^ T cell function (12). T cell activation can lead to cell exhaustion (11, 13). Considering the advantages and disadvantages of HIV-1-specific CD8^+^ T cells in HIV-1 infection, identifying some unconventional CD8^+^ T cell subsets that can specifically and effectively inhibit HIV-1 progression and reduce HIV-1 reservoir size will help in the management of HIV-1 infection.

CD11c, as integrin α_x_ and a commonly used marker of dendritic cells (DCs), is normally expressed on CD8^+^ T cells (14). This was first identified in autoimmune disease models (15). A subsequent study reported that CD8^+^ T cell activation coincided with CD11c upregulation, and that the majority of the tumor-infiltrating CD8^+^ T cells were CD11c^+^ in ovalbumin and poly(I:C)-treated mice, reflecting tumoricidal efficacy (16). Jamal et al. reported that number of CD11c^+^ CD8^+^ T cells increased in the blood and cervical tissues of mice and women with genital tract infections (17). Moreover, peripheral blood had more CD11c^+^ CD8^+^ T cells than primary lymphoid organs following acute virus infection (18). This subset displayed an effector function with higher activation and stronger secretory and cytotoxic capacity compared to CD11c^-^ T cells, aiding in the elimination of pathogenic microbes in mouse models (19). CD11c expression on CD8^+^ T cells can be induced by infection or vaccination and is associated with activated antigen-specific T cells, as demonstrated *in vitro* and *in vivo* (19, 20). Although CD11c^+^ CD8^+^ T cells may be a potential therapeutic target for combating acute infection and tumors in mouse models, the anti-HIV-1 activity of CD11c^+^ CD8^+^ T cells in patients with chronic HIV-1 infection remains unclear.

In this study, healthy controls (HC), HIV-1-infected treatment-naïve patients (TN), and HIV-1-infected patients treated by ART (ART) were enrolled to evaluate the role of CD11c^+^ CD8^+^ T cells in controlling HIV-1 infection. CD11c^+^ CD8^+^ T cells had a higher activation state and higher secretory and cytotoxic capacity with a lower level of exhaustion marker in controlling HIV-1 infection, indicating a potential treatment efficacy in HIV-1-infected patients.

## Materials and Methods

### Study Participants

Peripheral blood samples were obtained from 63 individuals from the Fifth Medical Center of Chinese PLA General Hospital. The participants included 30 TNs, 16 ARTs (achieved sustained viral suppression for >2 years and CD4 count > 350 cells/μl), and 17 HCs (detailed clinical data of the subjects are shown in **Table 1**). Peripheral blood mononuclear cells (PBMCs) were isolated by density gradient centrifugation using Ficoll-Paque PLUS (GE Healthcare, Piscataway, NJ, USA). Written informed consent was obtained from all the study participants. This study was performed in accordance with the Declaration of Helsinki and was approved by the Research Ethics Committee of the Fifth Medical Center of Chinese PLA General Hospital.

**Table 1:** Demographic characteristics of the participants for blood samples.

Characteristics	HCs (n = 17)	TNs (n = 30)	ARTs (n = 16)
Gender (male/female)	15/2	28/2	15/1
Age (years)	28 (27-30)	29 (23-37.75)	33 (26.5-37.25)
CD4^+^ T (cells/μl)	788 (504.5-932.5)	375 (282-447.8)	633 (561-836.8)
CD8^+^ T (cells/μl)	622 (463.5-877.5)	1062 (769-1396)	790.5 (632.3-1107)
CD4/CD8 ratio	1.10 (0.87-1.62)	0.37 (0.26-0.45)	0.83 (0.53-1.21)
Plasma viral load (log_10_ copies/ml)	NA	3.75 (3.25-4.52)	< 80 copies/ml
ART time (months)	0	0	55.5 (40.25-73.25)

All indicators except gender are shown as median (interquartile range); NA, not acquired.

### Flow Cytometry

For phenotypic staining, PBMCs were stained with the following antibodies: anti-HIV-1 pentamer-PE (HIV-1 gag p17 76-84R, HIV-1 gag gp41 67-75R, and HIV-1 nef 72-82R; Proimmune, Oxford, UK); anti-CD11c PE (BD Biosciences, Franklin Lakes, NJ, USA); anti-CD45-RA PE and anti-CD38 PE-Cy7 (eBioscience, Waltham, MA, USA); and anti-CD3 APC-Cy7, anti-CD4 APC-Cy7, anti-CD8 BV510, anti-CD8 Percp, anti-CD11c FITC, anti-CCR7 Percp, anti-HLA-DR BV421, and anti-PD-1 APC (BioLegend, San Diego, CA, USA). For intracellular cytokine staining, PBMCs were stimulated with anti-CD3 (Peprotech, Rocky Hill, NJ, USA, 1 µg/ml) and anti-CD28 (R&D, Minneapolis, MN, USA, 1 µg/ml) for 8 h at 37°C in the presence of 5% CO_2_. Brefeldin A (BD Biosciences) and CD107a APC (eBioscience) were added in the last 6 h. Cells were permeabilized using a Cytofix/Cytoperm Kit (BD Biosciences) and stained with the following antibodies: anti-IFN-γ PE-Cy7, anti-IL-2 PE, anti-TNF-α BV421, anti-granzyme-B PE, and anti-perforin PE-Cy7 (BioLegend). For HIV-1-specific CD8^+^ T cell evaluation, PBMCs were stained with anti-HIV pentamer-PE for 1 h, then stained with anti-CD4 and anti-CD8 antibodies at 37°C for 20 min following stimulation for 16 h with anti-CD3/CD28 (1 µg/ml) or HIV-1 overlapping peptides (HIV-1 pol, gag, and env; JPT, Berlin, Germany). The data were obtained using FACS-Canto (BD Biosciences) and analyzed using FlowJo version X (FlowJo, Ashland, OR, USA). Detailed information on all antibodies used is provided in **Supplementary Table 1**.

### Detection of HIV-1 DNA/Unspliced RNA (usRNA)

Total cellular DNA and usRNA were extracted from PBMCs using the QIA symphony DNA Mini kit (Qiagen, Valencia, CA, USA) and HiPure Total RNA Plus Mini Kits (Magen, Guangzhou, China), respectively. The HIV-1 DNA/usRNA ratio was determined using the HIV-1 Quantitative Detection Kit (SUPBIO, Guangzhou, China) according to the manufacturer’s instructions.

### Cell Sorting of CD11c^+^ and CD11c^-^ CD8^+^ T Cells

CD8^+^ T cells were purified from PBMCs using magnetic-activated cell sorting beads (130–093-227; Miltenyi Biotec, Bergisch-Gladbach, Germany) according to the manufacturer’s instructions. After labeling CD8^+^ T cells with anti-CD3-APC/Fire750, anti-CD8-BV510, and anti-CD11c-FITC antibodies, CD11c^+^ and CD11c^-^ CD8^+^ T cells were sorted using a MA900 Cell Sorter (Sony Biotechnology, Tokyo, Japan).

### RNA Purification and Microarray Analysis

According to the manufacturer’s instructions, total RNA from the sorted CD11c^+^ and CD11c^-^ CD8^+^ T cells was extracted using the PicoPure RNA Isolation Kit (Applied Biosystems, Foster City, CA, USA). Whole transcriptome amplification and tag-based library preparation were conducted using SMART-seq2, and sequencing was performed using the NextSeq 500 platform (Illumina, San Diego, CA, USA) by Annoroad (Beijing, China). RSEM (v1.2.22) supported by STAR aligner software (STAR 2.5.1b) was used to quantify transcript abundance and compare with the GRCh38 human genome assembly. The upper quantile normalization method was then used to normalize the parts per million transcripts in all the samples. In the differential expression analysis, genes with ratios > 2, *P* < 0.05, and false discovery rate (FDR) < 0.05, were considered to be significant. In Gene Ontology (GO) and Kyoto Encyclopedia of Genes and Genomes (KEGG) analyses, terms with *P* < 0.05 were considered significant.

### Killing Assay

To investigate the killing capacity of CD11c^+^ CD8^+^ T cells or CD11c^-^ CD8^+^ T cells, each purified cell type was co-cultured with Raji-bal-luc cells labeled with HIV-1 bal and luciferase (target group) and corresponding Raji-bal-luc cells (control group) for 6 h, respectively. Supernatants were discarded and cells were resuspended in substrate solution for 5 min. Luciferase signals were measured using a Spark multimode microplate reader (Tecan, Männedorf, Switzerland). Killing rates were calculated as the ratio of the difference between the target and control groups to the control group.

### Statistical Analyses

Statistical analyses were performed using GraphPad Prism 8.0 (GraphPad Software, San Diego, CA, USA). The Mann–Whitney U-test (unpaired) and Wilcoxon paired test (paired) were used to compare median values between the two groups. The Friedman test (unpaired) and Kruskal–Wallis (paired) tests were used to compare median values among three or more groups. The Pearson correlation coefficient was used to assess the correlation between two variables. In all analyses, *P* < 0.05 was considered statistically significant.

## Results

### CD11c^+^ CD8^+^ T Cells Show Transcriptional Characteristics of An Activated Effector T Cell Population

**Figure 1:**
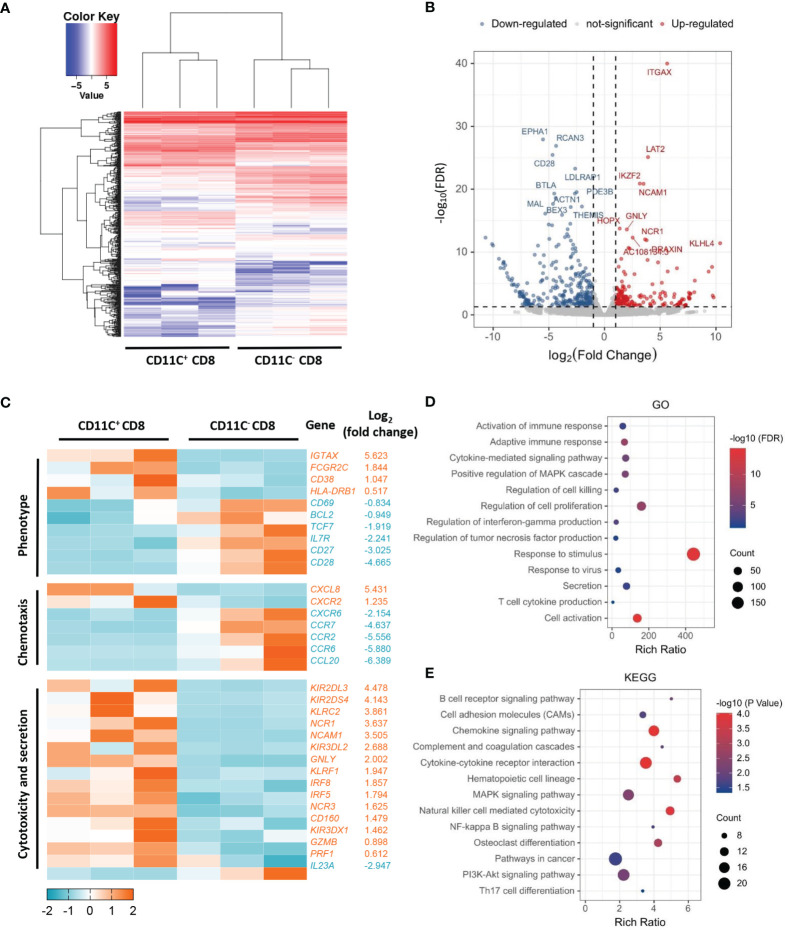
Distinct transcriptome profiles of CD11c^+^ and CD11C^-^ CD8^+^ T cells. **(A)** Heatmap of transcriptomic profiling of CD11c^+^ and CD11c^-^ cell subsets. **(B)** Volcano plot of differentially expressed genes. Rich ratio > 2, *P* < 0.05, FDR < 0.05 were considered significant. **(C)** Heatmap of representative genes with the log_2_ (fold change). **(D)** GO and **(E)** KEGG enrichment analyses showing the significantly enriched (*P* < 0.05) terms.

To understand the differences between CD11c^+^ CD8^+^ and CD11c^-^ CD8^+^ T cells in human PBMCs, we isolated the two cell populations from three healthy donors and analyzed their gene expression profiles by microarray analyses. The overall patterns of gene expression in the two cell populations are shown in **Figure 1A**. The downregulated and upregulated differentially expressed genes (*CD28* and *GNLY*, respectively) are shown in **Figure 1B**. Clustering analysis revealed that CD11c^+^ CD8^+^ T cells expressed higher levels of genes involved in T cell activation (*CD38* and *HLA-DR*), cytotoxicity (*GNLY*, *GZMB*, and *PRF1*), IFN-signaling (*IRF8* and *IRF5*), and lower levels of memory-related genes (*CD28, IL-7R*, and *CD27*) compared to CD11c^-^ CD8^+^ T cells (**Figure 1C**). Moreover, CD11c^+^ CD8^+^ T cells were highly enriched in transcripts of genes associated with activation of immune response, adaptive immune response, cytokine-mediated signaling pathway, regulation of cell killing, response to virus and stimulus, cytokine secretion, and mitogen-activated protein kinase (MAPK) and phosphoinositide 3-kinase (PI3K)-Akt signaling pathway, as demonstrated by GO and KEGG pathway analyses (**Figures 1D, E**). The collective findings indicate that CD11c^+^ CD8^+^ T cells may be a multifunctional effector subset.

### Increased Frequency of CD11c^+^ CD8^+^ T Cells Negatively Correlates With Disease Progression in HIV-1-Infected Patients

**Figure 2:**
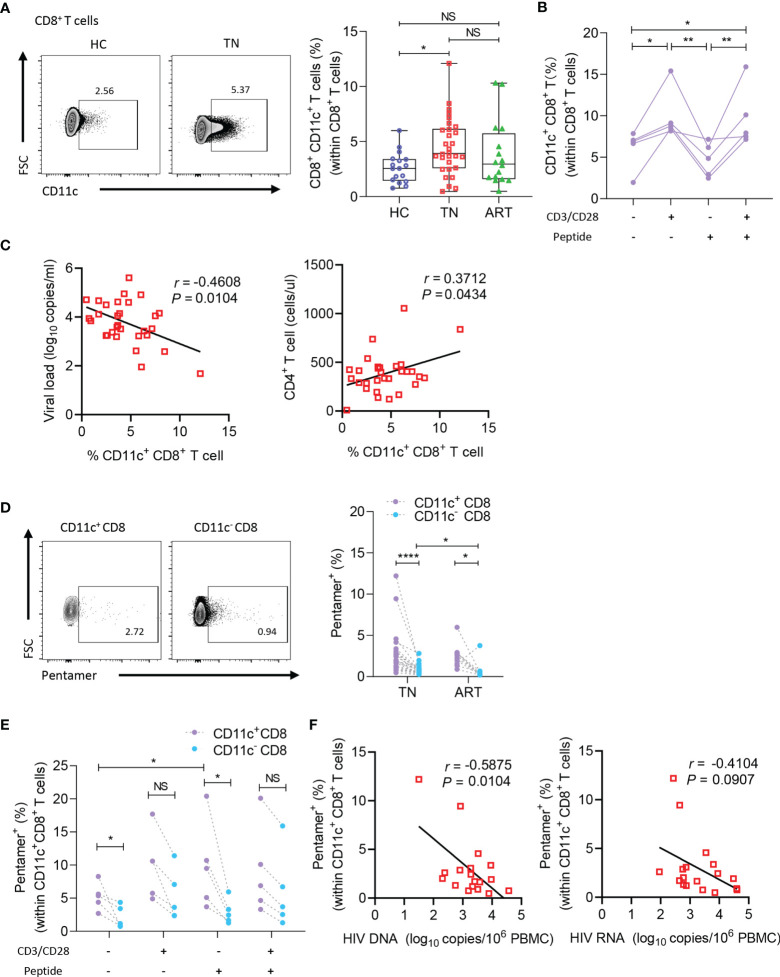
CD11c^+^ CD8^+^ T cells increase in number and negatively correlate with disease progression in HIV-1-infected patients. **(A)** Representative flow cytometry plots and the frequencies of circulating CD11c^+^ CD8^+^ T cells in HCs (n = 17) and TN (n = 30) and ART (n = 34) patients. **(B)** Frequency of CD11c^+^ CD8^+^ T cells induced by CD3/CD28 or HIV-1-derived overlapping peptides. **(C)** Correlation between the frequency of circulating CD11c^+^ CD8^+^ T cell with plasma HIV-1 viral load and CD4^+^ T cell count (n = 30). **(D)** Representative flow cytometry plots and the frequencies of HIV-1-pentamer^+^ CD11c^+^ CD8^+^ T cells in PBMCs from TN (n = 22) and ART (n = 22) patients. **(E)** Frequency of HIV-1-pentamer^+^ CD11c^+^ CD8^+^ T cells induced by CD3/CD28 or HIV-1-derived overlapping peptides. **(F)** Correlation between the frequency of HIV-1-pentamer^+^ CD11c^+^ CD8^+^ T cells with HIV-1 DNA and HIV-1 usRNA (n = 18). The numbers in representative flow cytometry plots indicate the percentage of gated cells. *P < 0.05; **P < 0.01; ****P < 0.0001; NS, not significant.

As CD11c^+^ CD8^+^ T cells are a potential multifunctional effector subset in humans, we enrolled HC, TN, and ART groups to explore the roles of CD11c^+^ CD8^+^ T cells during chronic HIV-1 infection. As illustrated in representative flow cytometry images of CD11c^+^ CD8^+^ T cells (**Figure 2A**, left), among the CD8^+^ T cells, frequencies of CD11c^+^ CD8^+^ T cells were higher in the TN group than in the HC group (**Figure 2A**, right). The findings suggested that CD11c^+^ CD8^+^ T cells increased in number upon HIV-1 infection. Additionally, we assessed the roles of CD3/CD28 and HIV-1-derived overlapping peptides in the induction of CD11c^+^ CD8^+^ T cells *in vitro*. As shown in **Figure 2B**, the frequencies of CD11c^+^ CD8^+^ T cells were strongly induced after CD3 and CD28 stimulation, but not after HIV-1-derived overlapping peptide. The frequencies of CD11c^+^ CD8^+^ T cells among CD8^+^ T cells were negatively correlated with plasma HIV-1 RNA levels (r = -0.3415; *P* = 0.0481), whereas the subset was positively correlated with CD4^+^ T cell count (r = 0.3702; *P* = 0.0441) (**Figure 2C**). We further determined the HIV-1-specific CD8^+^ T cells among the CD11c^+^ CD8^+^ T cells, as delineated in the representative dot plots of pentamer^+^ CD8^+^ T cells (**Figure 2D**, left). The frequencies of HIV-1-specific CD8^+^ T cells among the CD11c^+^ CD8^+^ T cells were remarkably higher in both TN and ART groups than in CD11c^-^ CD8^+^ T cells. The frequencies of HIV-1-specific CD11c^-^ CD8^+^ T cells were significantly reduced during ART, but the decrease in HIV-1-specific CD11c^+^ CD8^+^ T cells was not significant (**Figure 2D**, right). In addition, this HIV-1-specific subset was more strongly induced than the CD11c^-^ counterparts by the stimulation of HIV-1-derived overlapping peptides, but not by CD3 and CD28 (**Figure 2E**). Furthermore, an inverse correlation was evident between the frequencies of HIV-1-specific CD8^+^ T cells among the CD11c^+^ CD8^+^ T cells and HIV-1 DNA levels in the TN group (r = -0.5920; *P* = 0.0076) (**Figure 2F**). Taken together, these findings suggest that HIV-1 infection induces the expansion of CD11c^+^ CD8^+^ T cells, especially HIV-1-specific CD8^+^ T cells among the CD11c^+^ CD8^+^ T cells, which are negatively associated with disease progression.

### High Activation and Low PD-1 Expression of CD11c^+^ CD8^+^ T Cells During Chronic HIV-1 Infection

**Figure 3:**
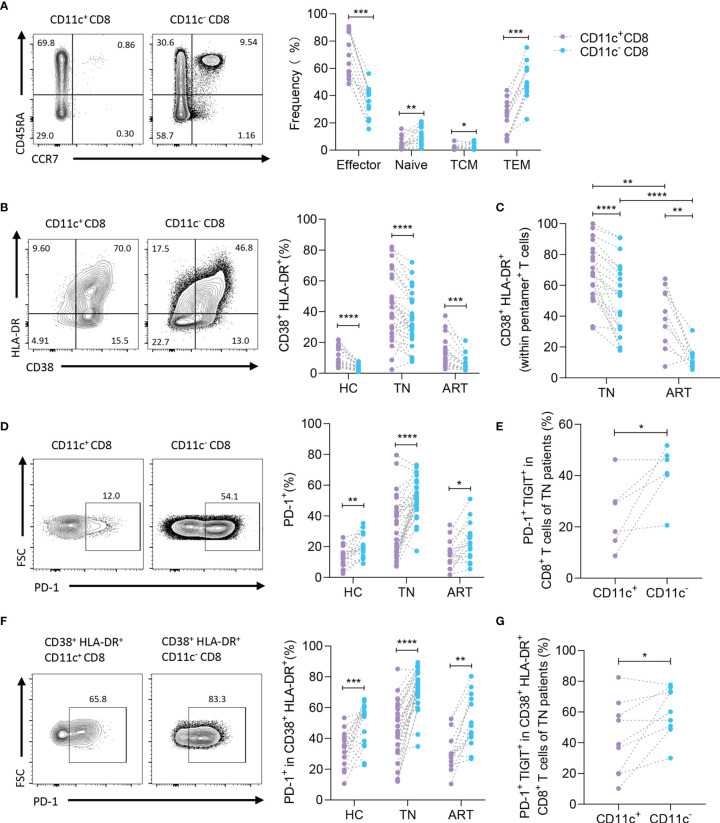
CD11c^+^ CD8^+^ T cells show higher activated and lower exhausted phenotypes. **(A)** Representative flow cytometry plots and the frequencies of effector (CCR7^-^ CD45RA^+^), naïve (CCR7^+^ CD45RA^+^), central memory (CCR7^+^ CD45RA^-^) (T_CM_), and effective memory (CCR7^-^ CD45RA^-^) (T_EM_) CD8^+^ T cell subsets. **(B)** Representative flow cytometry plots and the frequencies of activated (CD38^+^ HLA-DR^+^) CD8^+^ T cells in CD11c^+^ and CD11c^-^ CD8^+^ T cells. **(C)** Frequencies of activated HIV-1-specific CD8^+^ T cells in CD11c^+^ and CD11c^-^ CD8^+^ T cells. **(D)** Representative flow cytometry plots of PD-1 expression (left panel), and frequency of PD-1^+^ T cells among the CD11c^+^ and CD11c^-^ CD8^+^ T cells (right panel). **(E)** PD-1^+^ TIGIT^+^ T cells in CD11c^+^ and CD11c^-^ CD8^+^ T cells. **(F)** Frequency of PD-1 and **(G)** PD-1^+^ TIGIT^+^ in CD38^+^ HLA-DR^+^ CD11c^+^ and CD11c^-^ CD8^+^ T cells. The numbers indicate the percentage of gated cells in representative flow cytometry plots. *P < 0.05; **P < 0.01; ***P < 0.001; ****P < 0.0001.

To determine the subtype of CD11c^+^ CD8^+^ T cells in HIV-1-infected patients, CD3, CD8, CD45RA, and CCR7 were detected as previously reported (**Figure 3A**, left) (21). As predicted, a large proportion of CD11c^+^ CD8^+^ T cells were considered effectors (CD45RA^+^ CCR7^-^ cells), whereas CD11c^-^ CD8^+^ T cells were mainly considered as effector or effector memory populations in the TN group (**Figure 3A**, right). Because HIV-1-mediated immune activation promotes CD8^+^ T cell exhaustion, particularly HIV-1-specific CD8^+^ T cells, and PD-1 expression on HIV-1-specific T cells is the leading marker of exhaustion associated with HIV-1-specific T cell dysfunction and disease progression (12, 22, 23), we first monitored the expression of activation markers (HLA-DR and CD38) to compare the activation profiles of CD11c^+^ CD8^+^ T cells and CD11c^-^ CD8^+^ T cells (**Figure 3B**) (24). Higher levels of HLA-DR and CD38 were detected in both CD11c^+^ CD8^+^ T cells and HIV-1-specific CD8^+^ T cells among the CD11c^+^ CD8^+^ T cell subset than in the corresponding T cells (**Figures 3B, C**). Next, we analyzed PD-1 expression on CD11c^+^ CD8^+^ T cells and CD11c^-^ CD8^+^ T cells in the HC, TN, and ART groups (**Figure 3D**, left). Lower levels of PD-1 were observed in CD11c^+^ CD8^+^ T cells compared to CD11c^-^ CD8^+^ T cells in all three groups (**Figure 3D**, right). We further observed that the co-expression of PD-1 and TIGIT was markedly lower in CD11c^+^ CD8^+^ T cells than in CD11c^-^ CD8^+^ T cells in TN group (**Figure 3E**). Based on the above findings, we further analyzed the expression of PD-1 and TIGIT in activated (CD38^+^ HLA-DR^+^) CD11c^+^ CD8^+^ and CD11c^-^ CD8^+^ T cells. Notably, compared to activated CD11c^-^ CD8^+^ T cells, PD-1 expression in the HC, TN, and ART groups and co-expression of PD-1 with TIGIT in the TN group were significantly lower in activated CD11c^+^ CD8^+^ T cells (**Figures 3F, G**). All these observations indicate that CD11c^+^ CD8^+^ T cell subset is the main effective factor among CD8^+^ T cells with high activation and low exhaustion in HIV-1 infection.

### Robust Antiviral Effect of CD11c^+^ CD8^+^ T Cells

**Figure 4:**
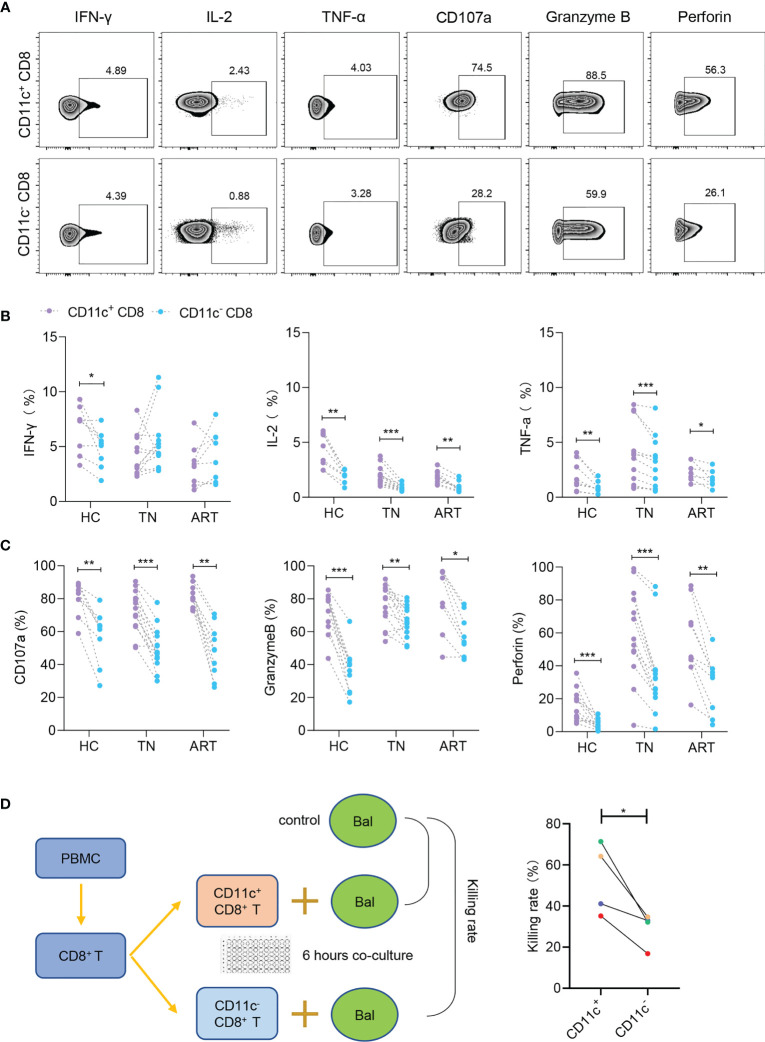
CD11c^+^ CD8^+^ T cells have strong secretory and cytotoxic capacity during HIV-1 infection. **(A)** Representative flow cytometry plots showing the staining of indicated cytokines and cytotoxicity-related molecules. The numbers indicate the percentage of gated cells. Expression of cytokines **(B)** and cytotoxicity-related molecules **(C)** in CD11c^+^ and CD11c^-^ CD8^+^ T cells. **(D)** Schematic of killing assay and killing rates of CD11c^+^ and CD11c^-^ CD8^+^ T cells to target cells. *P < 0.05; **P < 0.01; ***P < 0.001.

Since the above results revealed that CD11c^+^ CD8^+^ T cells were a highly activated population with lower PD-1 expression, we further investigated the functional signatures of secretion (IFN-γ, IL-2, and TNF-α) and cytotoxicity (CD107a, granzyme B, and perforin) during HIV-1 infection (**Figure 4A**). The levels of IL-2 and TNF-α were higher in CD11c^+^ CD8^+^ T cells than in CD11c^-^ CD8^+^ T cells among all the groups. However, IFN-γ secretion was impaired in CD11c^+^ CD8^+^ T cells in the TN and ART groups (**Figure 4B**). CD11c^+^ CD8^+^ T cells expressed higher levels of CD107a, granzyme B, and perforin than CD11c^-^ CD8^+^ T cells among all subjects (**Figure 4C**). CD11c^+^ CD8^+^ T cells also showed a significantly higher killing rate (%) for Raji-bal-luc cells compared to CD11c^-^ CD8^+^ T cells, indicating the strong antiviral capacity of CD11c^+^ CD8^+^ T cells against HIV-1 (**Figure 4D**). Collectively, these results suggest that, in contrast to CD11c^-^ CD8^+^ T cells, CD11c^+^ CD8^+^ T cells are multifunctional, with higher expression of secretory (IL-2 and TNF-α) and cytotoxic (CD107a, granzyme B, and perforin) markers.

## Discussion

Although CD8^+^ T cells, particularly HIV-1-specific CD8^+^ T cells, play a crucial role in the elimination of HIV-1 (25, 26), exhausted CD8^+^ T cells with functional defects develop during chronic HIV-1 infection and are linked to clinical progression (11, 12). In this study, we observed a dramatic increase in the frequency of CD11c^+^ CD8^+^ T cells. This increase was negatively associated with disease progression, and a higher frequency of HIV-1-specific CD8^+^ T cells among the CD11c^+^ CD8^+^ T cells was negatively associated with HIV-1 DNA levels. Interestingly, unlike conventional CD8^+^ T cells characterized by high activation along with high PD-1 expression (27), CD11c^+^ CD8^+^ T cells were highly activated and expressed low levels of PD-1 in all three study groups. Additionally, this subset expressed higher cytotoxic markers (granzyme B, perforin, and CD107a) and cytokines (IL-2 and TNF-α) and displayed stronger cytotoxicity against HIV-antigen-labeled cell lines than CD11c^-^ CD8^+^ T cells. The collective findings indicate that CD11c^+^ CD8^+^ T cells are rich in an HIV-specific subgroup with high activation, low exhaustion, and strong killing ability, which may play an important role in the control of HIV-1 infection.

A previous study demonstrated that CD11c^+^ CD8^+^ T cells increased in number following the challenge of microbes, such as with Herpes Simplex Virus type 1 (HSV-1) and malaria parasite (16, 19, 20, 28) in animal models and that this subset contributes to the reduction of viral titers *in vivo* (28). In this study, we also found that CD11c^+^ CD8^+^ T cell counts were increased in chronic HIV-1-infected TN patients and were negatively correlated with viral load and positively associated with CD4^+^ T cell count in these patients. HIV-specific CD8^+^ T cell populations can effectively control viral replication (29). Consistent with this, we further found that HIV-1-specific CD8^+^ T cells were more enriched in CD11c^+^ CD8^+^ T cells than in CD11c^-^ subsets and were inversely correlated with HIV-1 DNA levels. Several studies have revealed that antigen-specific CD11c^+^ CD8^+^ T cells can be robustly induced by antigen re-challenge derived from memory CD8^+^ T cells (20), and that CD11c^+^ CD8^+^ T cells are a subset of antigen-specific T cells with potent antiviral effects *in vivo* (16, 19, 28). In the present study, *in vitro* experiments showed that CD11c^+^ CD8^+^ T cell counts were significantly increased after stimulation with CD3 and CD28, rather than HIV-1-derived overlapping peptides. In contrast, HIV-specific CD11c^+^ CD8^+^ T cells could be induced by HIV-1-derived overlapping peptides, marking a population of HIV-specific subgroups. Collectively, these results highlight the potential role of CD11c^+^ CD8^+^ T cells, especially the virus-specific subset, as a novel therapeutic factor for HIV-1 infection.

Previous studies have reported that the expression of exhaustion markers, such as PD-1, in CD8^+^ T cells is affected by persistent exposure to high levels of antigens and immune activation (11, 23). T cell exhaustion leads to functional defects, including impaired cytokine production and cytotoxic capacity associated with HIV-1 plasma viral load and CD4^+^ T cell count (11, 30, 31). Marc et al. reported that CD11c^+^ CD8^+^ T cells were highly activated with higher expression of CD69 and CD54 compared to CD11c^-^ CD8^+^ T cells in an acute respiratory syncytial virus (RSV)-infected mouse model (28). Consistent with these results, we too found that CD11c^+^ CD8^+^ T cells were highly activated. However, unlike the conventional CD8^+^ T cells, activated CD11c^+^ CD8^+^ T cells expressed low levels of PD-1 in all groups. The low expression of PD-1 may in turn rescue CD8^+^ T cell function, such as activation, proliferation, and cytokine secretion by inhibiting basic leucine zipper transcription factor, ATF-like (BATF) expression (9). According to our results, lower levels of PD-1 on highly activated CD11c^+^ CD8^+^ T cells in chronic HIV-1-infected patients may make the subset a potent effector to combat HIV-1; the underlying molecular mechanism merits further research.

Since effective control of HIV-1 was predominantly mediated by effector CD8^+^ T cells rather than antibodies (32), we further confirmed that CD11c^+^ CD8^+^ T cells expressed higher levels of cytokines (IL-2 and TNF-α) compared to CD11c^-^ CD8^+^ T cells in chronic HIV-1-infected patients. These findings are consistent with the findings of the subset in malaria parasite, tumor-implant mouse model, and RSV-infected mouse model (16, 19, 28). Unlike these studies, the higher expression of IFN-γ on CD11c^+^ CD8^+^ T cells compared with that on CD11c^-^ CD8^+^ T cells was only observed in the HC group, which might indicate impaired IFN-γ production in HIV-1-infected patients due to chronic infection. CD11c^+^ CD8^+^ T cells demonstrated a higher expression level of cytotoxicity-related genes and a higher cell killing rate against Raji-Env cells *in vitro* compared to CD11c^-^ CD8^+^ T cells, suggesting a stronger targeted cell cytotoxicity of CD11c^+^ CD8^+^ T cells. This is consistent with previous studies (16, 19, 28). This efficient antiviral activity of CD11c^+^ CD8^+^ T cells may be attributed to the fact that the subset had characteristics of high activation and HIV-1 specificity with low levels of PD-1 during chronic HIV-1 infection. This evidence distinguishes CD11c^+^ CD8^+^ T cells from conventional activated effector T cell subsets, making it a potential option for HIV-1 immunotherapy.

This study had two main limitations. Firstly, CD11c^+^ CD8^+^ T are significantly amplified in peripheral blood and multiple tissues in mice during acute virus infection (18). However, there is still a lack of data on the amplification of these cells in human tissues. We should pay additional attention to the distribution and phenotype of CD11c^+^ CD8^+^ T cells in the tissues of HIV-1-infected patients, rather than a particular cell population. Secondly, the role and mechanism of CD11c in CD8^+^ T cells is still unknown and requires further investigation.

In summary, we demonstrate a HIV-1-specific CD11c^+^ CD8^+^ T cell subgroup with characteristics of high activation, low PD-1 expression, and strong cytotoxicity. The subgroup is critical in the control of chronic HIV-1 infection. Future studies should evaluate the immunotherapy efficacy of CD11c^+^ CD8^+^ T cells in eliminating HIV-1 reservoirs in clinical trials.

## Data Availability Statement

The datasets presented in this study can be found in online repositories. The names of the repository/repositories and accession number(s) can be found below: https://www.ncbi.nlm.nih.gov/geo/ GSE183022.

## Ethics Statement

The studies involving human participants were reviewed and approved by the Fifth Medical Center of PLA General Hospital Research Ethics Committee. The patients/participants provided their written informed consent to participate in this study.

## Author Contributions

F-SW, J-FZ, and Y-MJ conceived the study and wrote the paper with A-LG. Y-MJ, A-LG, and J-FZ designed and performed most experiments. H-HH, Y-MJ, and J-FZ contributed to the clinical samples and data collecting. A-LG, J-FZ, and LG performed statistical and flow cytometry analysis. J-YZ, CZ, J-WS, R-NX, XF, and MS contributed to scientific planning. Intellectual input was provided by all authors. All authors contributed to the article and approved the submitted version.

## Funding

This study was supported by the Innovation Groups of the National Natural Science Foundation of China (grant no. 81721002), Peking University Clinical Scientist Program Special (grant no. BMU2019LCKXJ013).

## Conflict of Interest

The authors declare that the research was conducted in the absence of any commercial or financial relationships that could be construed as a potential conflict of interest.

## Publisher’s Note

All claims expressed in this article are solely those of the authors and do not necessarily represent those of their affiliated organizations, or those of the publisher, the editors and the reviewers. Any product that may be evaluated in this article, or claim that may be made by its manufacturer, is not guaranteed or endorsed by the publisher.
