# Supplementary material for: HIV-1-Specific CD11c+ CD8+ T Cells Display Low PD-1 Expression and Strong Anti-HIV-1 Activity
Source: Front Immunol. 2021 Oct 15;12:757457. doi: 10.3389/fimmu.2021.757457 (PMC8554207; doi:10.3389/fimmu.2021.757457)
Supplement: Supplementary file 1 [file Table_1.docx]

Supplementary Material

**Supplementary Table 1.** Antibodies used in this study.

| Reagent or resource | Source | Clone | Catalogue number |
| --- | --- | --- | --- |
| Anti-human CD3 APC/Fire750 | Biolegend | SK7 | 344840 |
| Anti-human CD3 APC-Cy7 | Biolegend | SK7 | 344818 |
| Anti-human CD4 APC-Cy7 | Biolegend | OKT4 | 317418 |
| Anti-human CD8 BV510 | Biolegend | SK1 | 344732 |
| Anti-human CD8 Percp | Biolegend | RPA-T8 | 301032 |
| Anti-human CD11c FITC | Biolegend | 3.9 | 301604 |
| Anti-human CD11c PE | BD | S-HCL-3 | 347637 |
| Anti-human CCR7 Percp | Biolegend | G043H7 | 353220 |
| Anti-human CD45RA PE | eBioscience | HI100 | 12-0458-42 |
| Anti-human CD38 PE-Cy7 | Biolegend | HIT2 | 25-0389-42 |
| Anti-human HLA-DR BV421 | Biolegend | L243 | 307636 |
| Anti-human PD-1 APC | Biolegend | EH12.2H7 | 329908 |
| Anti-human GB PE | Biolegend | QA16A02 | 372208 |
| Anti-human Perforin PE-Cy7 | Biolegend | B-D48 | 353315 |
| Anti-human CD107a APC | eBioscience | eBioH4A3 | 50-1079-42 |
| Anti-human IFN-γ PE-Cy7 | Biolegend | B27 | 506518 |
| Anti-human IL-2 PE | Biolegend | MQ1-17H12 | 500307 |
| Anti-human TNF-α BV421 | Biolegend | MAb11 | 502932 |
